# Sensitive genotyping of mutations in the *EGFR* gene from NSCLC patients using PCR-GoldMag lateral flow device

**DOI:** 10.1038/s41598-017-08210-8

**Published:** 2017-08-21

**Authors:** Xian-Ying Li, Chao Zhang, Qin-Lu Zhang, Juan-Li Zhu, Qian Liu, Ming-Wei Chen, Xue-Min Yang, Wen-Li Hui, Ya-Li Cui

**Affiliations:** 1 College of Life Sciences, Northwest University, Xi’an, 710069 China; 2 School of Medicine, The First Affiliated Hospital of Xi’an Jiaotong University, Xi’an, 710061 China; 3 Shaanxi Provincial Engineering Research Center of Nano-Biomedical Detection, Xi’an, 710077 China

## Abstract

Epidermal growth factor receptor (*EGFR*) mutations predict better outcomes with *EGFR* tyrosine kinase inhibitors in patients with non-small cell lung cancer (NSCLC). Most common activating mutations include in-frame deletion in exon 19 and L858R substitution in exon 21, which account for >90% of all *EGFR* mutations in NSCLC. In this study, a PCR-GoldMag lateral flow assay (PCR-GoldMag LFA) was developed for the visual detection of delE746-A750 and L858R of *EGFR* mutations. Forty formalin-fixed paraffin-embedded (FFPE) tissue samples of NSCLC patients were analyzed using PCR-GoldMag LFA system and verified by direct sequencing and TaqMan-PCR detection methods. Results showed that *EGFR* mutations were detected in 34 cases among the 40 samples (85%) by PCR-GoldMag LFA method. Among the 34 cases, 5 cases were simultaneously detected with delE746-A750 in exon 19 and L858R mutation in exon 21. Compared with sequencing, only 4 samples were detected as delE746-A750, which revealed higher sensitivity of PCR-GoldMag LFA detection method than direct sequencing. TaqMan-PCR method verified the L858R mutation and was in 100% agreement with our method. These results indicated that our method has obvious advantages to analyze clinical samples and offers a more sensitive alternative to direct sequencing for the detection of *EGFR* mutations.

## Introduction

Lung cancer is one of the leading causes of death worldwide and is expected to remain a major health problem in the future^1^. Lung cancer is classified as non-small cell lung cancer (NSCLC) and small cell lung cancer (SCLC). Majority (75–85%) of lung cancer cases constitutes NSCLC^2^ and an individual therapy based on the genotype against NSCLC has been put forward. Therapeutic strategy using epidermal growth factor receptor (*EGFR*)-activating mutations was beneficial for patients who showed an increased response to an *EGFR*-TK inhibitor (e.g. gefitinib or erlotinib) treatment^3^. These mutations conferred sensitivity to tyrosine kinase inhibitors (TKIs) and are found within exons 18 ~ 21 of the TK domain of *EGFR* ^4, 5^. Approximately 90% of all activating *EGFR* mutations include short in-frame deletion in exon 19 (delE746-A750) and a specific mutation in exon 21 (L858R)^6^. These two mutations are closely correlated with good response to TKIs treatment in patients with NSCLC^7–9^. Therefore, testing of *EGFR* mutations has become a very important step in the treatment-response to the drugs before undertaking any therapeutic decision^10^.

Recently, a number of genotyping methods have been developed to detect the deletion and point mutations in the *EGFR* gene. Although direct sequencing is the gold standard for detection of *EGFR* mutations, it demonstrates low sensitivity and needs improvement in the turnaround time for routine diagnosis. Therefore, the next-generation sequencing and pyrosequencing^11^, denatured high-performance liquid chromatography (dHPLC)^12, 13^ and high-resolution melting analysis (HRMA) techniques^14, 15^ have been used as “screening methods” to detect all types of mutations including novel variants. The PCR-based methods were assigned as the “targeted method” for deletions in exon 19 and the L858R point mutation in exon 21. Because of its improved sensitivity, RT-PCR^16, 17^ is recommended for the detection of mutations compared to other methods like direct sequencing. Several new techniques, such as Smart Amplification Process (Smart AMP)^18, 19^, CCP-based FRET^20^ have also been used for the target detection. However, these “target methods” are relatively expensive, time consuming or invariably require favorable experimental conditions and sophisticated instruments. Amplification refractory mutation system (ARMS)-PCR is a simple and accurate method that could discriminate between mutant and wild-type DNA. GoldMag lateral flow device combined with ARMS-PCR was set up as a simple and rapid genotyping method for methylenetetrahydrofolate reductase (*MTHFR*) C677T^21^. The new PCR-GoldMag lateral flow device for SNP detection, including *MTHFR* C677T and Apolipoprotein E polymorphisms, has been validated by sequencing for more than 2,000 genomic DNAs in 6 hospitals in China, and showed a high specificity and sensitivity^21, 22^. This method allows for rare signals to be detected with greater sensitivity, tends to be faster and cheaper, and hence can be used as “targeted method” for genotyping of *EGFR* gene. Here, we first demonstrate a PCR-GoldMag LFA for the two most common therapy-related EGFR mutations, delE746-A750 and L858R.

## Results

### Principles of PCR-lateral flow assay

To detect the mutant sites of E746-A750, Bi-PASA-LFA method was established. The Bi-PASA technique in our study was used to amplify target fragments in two tubes instead of the traditional one tube. One was WT tube with primer A and primer Q, primer A and primer Q are 5′end-labelled with digoxin and biotin, respectively. A 325-bp AQ fragment was used to detect the wild genotype. The other was M tube with primer P and primer B. Similar to the WT tube primers, primer P and primer B are also 5′end-labelled with digoxin and biotin, respectively. A 155-bp PB fragment was used to detect the deletion genotype. Similarly, to detect L858R point mutant, we established ARMS-PCR-GoldMag LFA method. Forward (M and WT) and reverse (common) primers are 5′end-labelled with digoxin and biotin, respectively. Equal amount of tumor DNA was added to the above two PCR tubes. After PCR amplification, the products of two tubes are added on the sample pads of two PCR-GoldMag LFA strips separately. Expected PCR fragments are allowed to bind to the PGMNs-anti-digoxin antibody conjugates on the adjacent conjugate pad, forming DNA-PGMNs-anti-digoxin antibody complexes. These complexes flow along the strip, and then were captured by pre-immobilized streptavidin on the test line (T line) with a result of a red band. The excess PGMNs-anti-digoxin antibody conjugates is captured by goat anti-mouse IgG on the control line (C line), evidencing the work of the system.

The final test result of a tumor sample is a combinational visual presentation as per the color development on the T lines of both strips. For homozygous mutant sample, a distinct red band is observable on the T line of the strip used only for the M tube but not for the WT tube. In contrast, for wild-type sample, the red band shows up exclusively on the strip receiving the WT tube but not the M tube. However, when red bands with similar intensities are present on the T lines of both the strips, it indicates a heterozygous mutant sample.

### Establishment of PCR-GoldMag LFA system for detection of delE746-A750 and L858R mutations

Before testing the clinical samples, both allele-specific PCR assays were validated in known genomic samples (wild-type controls DNA, heterozygous mutant control DNA from cell lines NCI-H-1650 and NCI-H-1975, Plasmids containing delE746-A750 homozygous mutation and L858R homozygous mutation, respectively).

To improve the specificity and sensitivity of our method, we carefully optimized the PCR procedure, which included the designing of primers, the concentration of primers, annealing temperature and the PCR cycles. All primers were designed with high specificity. To determine the optimal concentration of the primers, a series of concentration gradient of each primer were set to use into the PCR mix. We also tested PCR cycles (e.g. for up to 26, 28, 30, 32, 34, 36 cycles). In order to obtain high sensitivity but no false-positive result, we choose 32 cycles as the optimal cycle number in our system. In addition, annealing temperatures were also tested (e.g. 56, 58, 60, 62, 64). Results showed that the optimal annealing temperature was set at 60 °C in our system.

#### Specificity of the PCR-GoldMag LFAs

**Figure 1:**
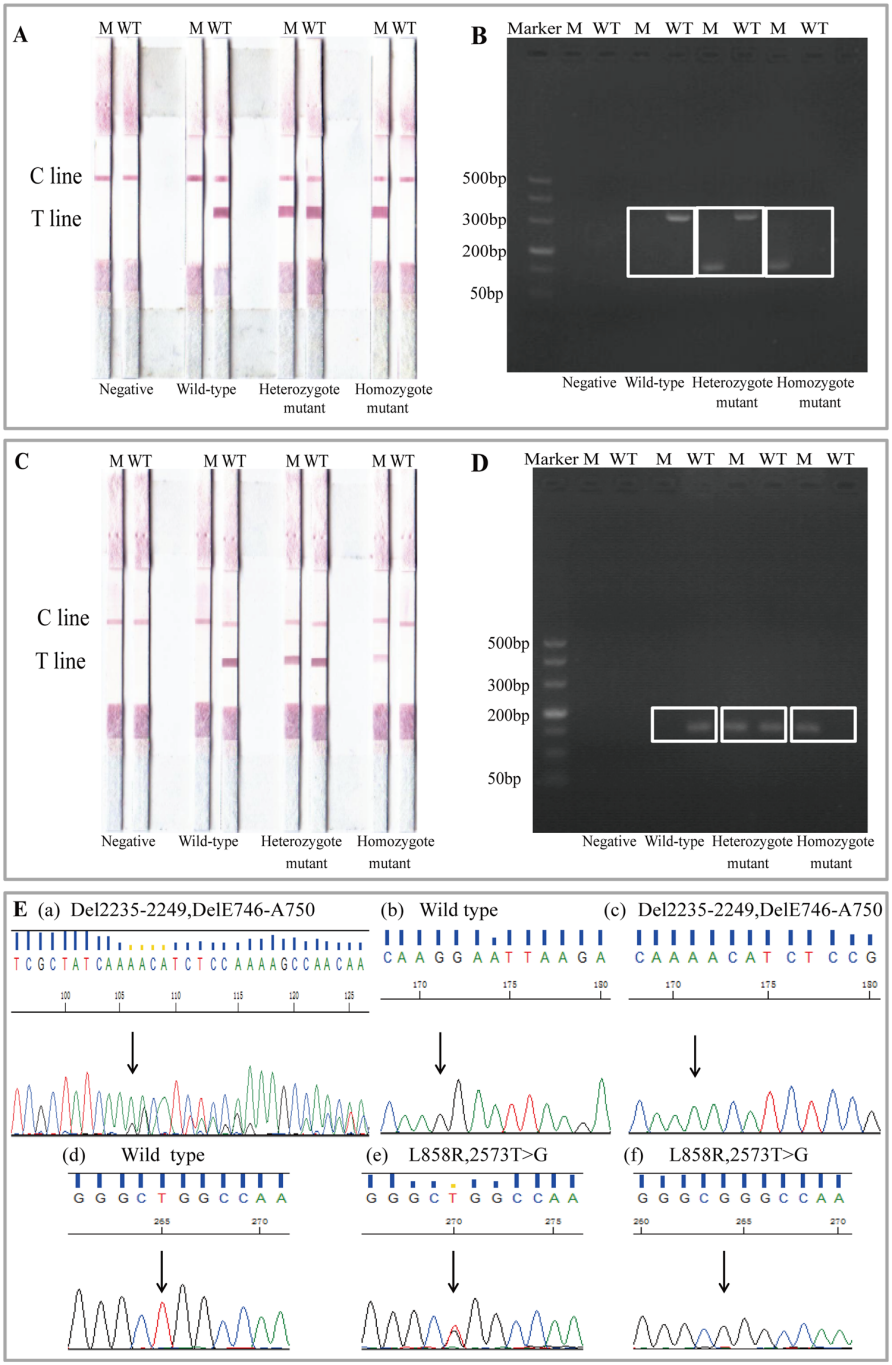
Evaluation of the specificity of E746-A750 and L858R detection using PCR-GoldMag LFAs. The test results of reference DNA samples (control DNA for wild type, cell line NCI-H-1650, NCI-H-1975 for heterozygous and plasmid containing delE746-A750, L858R for homozygous mutations) of E746-A750 (**A**) and L858R (**C**) by PCR-GoldMag LFA. (M = M tube. WT = WT tube.) Agarose gel electrophoresis results of E746-A750 (**B**) and L858R (**D**). (M = M tube. WT = WT tube.) (**E**) Sequencing results of E746-A750 and L858R. (a) 2235-2249del15bp detected in exon 19 from cell line NCI-H-1650. (b) Wild type of exon 19. (c) 2235-2249del15bp detected in exon 19 from plasmid. (d) Wild type sequence of L858R in exon 21. (e) 2573T > G detected in exon 21 in cell line NCI-H-1975. (f) 2573T > G detected in exon 21 in plasmid containing L858R homozygous mutation.

The specificity of *EGFR* mutation testing was a key part for the whole research. Known genomic samples (wild-type control DNA from healthy individuals, heterozygous mutant control DNA from cell lines NCI-H-1650 and NCI-H-1975, plasmids containing delE746-A750 homozygous mutation and L858R homozygous mutation, respectively) were used to validate the specificity of our method. All the known genomic samples and negative control were tested by PCR-GoldMag LFA system (Fig. 1A and 1C). Results of agarose gel electrophoresis were shown in Fig. 1B and 1D. To obtain high specificity, with no false positives, 32 cycles of PCR amplification will suffice for the PCR-GoldMag LFA detection. However, electrophoresis requires at least 35 cycles followed by a series of time-consuming steps. In addition, the testing results detected by sequencing are displayed as a comparison in Fig. 1E.

#### Sensitivity of the PCR-GoldMag LFAs

**Figure 2:**
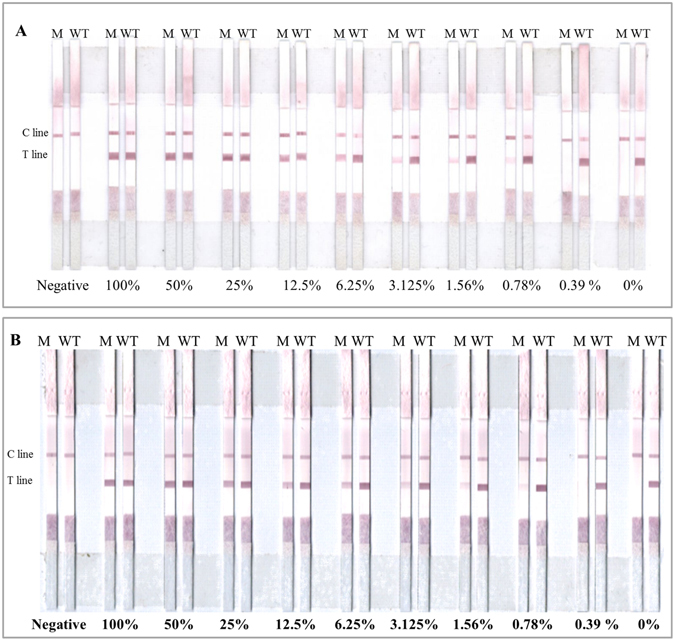
Sensitivity testing of the PCR-GoldMag LFA by serial dilution experiment. Serial dilution of the heterozygous mutant cell line DNA NCI-H-1650 and NCI-H-1975, with control wild-type DNA from cell lines NCI-A549, respectively. Mixtures with the following proportions of DNA from mutant cells were prepared: 100%, 50%, 25%, 12.5%, 6.25%, 3.125%, 1.56%, 0.78%, and 0% (no mutant DNA). (**A**) E746-A750 deletion. (**B**) The L858R mutation.

The sensitivity of our method was evaluated by applying the method to mixtures of DNA with varying ratios of mutant and wild-type DNA. The mutated DNA, isolated from tumor cell lines NCI-H-1650 (containing heterozygous mutations delE746-A750) and NCI-H-1975 (containing heterozygous mutations L858R point mutant), was mixed with various amounts of wild-type DNA from NCI-A549 cell lines, respectively. Mixtures with the following proportions of DNA from mutant cells were prepared: 100%, 50%, 25%, 12.5%, 6.25%, 3.125%, 1.56%, 0.78%, 0.39% and 0% (no mutant DNA). Using the PCR-GoldMag LFA assay, we were able to detect delE746-A750 and L858R mutations of the *EGFR* gene in the mixture with as low as 0.78% of mutant DNA, which suggested high sensitivity of our method (Fig. 2).

### Clinical sample testing

A total of 40 NSCLC FFPE specimens were detected using the PCR-GoldMag LFA assay. *EGFR* mutations were detected in 34 cases (85%, 34/40), which included E746-A750 deletion mutations in 5 cases (12.5%, 5/40), L858R point mutations in 34 cases (85%, 34/40), both delE746–A750 and L858R mutations in 5 cases. As a comparison, all samples were detected for E746-A750 deletion mutation with direct sequencing. Results showed that only 4 of the 40 (10%, 4/40) samples exhibited E746-A750 deletion mutation, one case harbors the L747-T751insP, and one case harbors L747–P753insS. TaqMan-PCR method was used to verify the L858R mutation. Thirty four samples with positive PCR-GoldMag LFA results were detected with L858R point mutations. The results were completely concordant with PCR-GoldMag LFA method (detail see Table 1).

**Table 1:** Genotyping results by PCR-GoldMag LFA, DNA sequencing (delE746-A750) and TaqMan-PCR (L858R) for 40 FFPE clinical samples.

Sample	Tumor	delE746-A750		L858R	
No.	type	PCR-GoldMag LFA	Direct sequencing	PCR-GoldMag LFA	TaqMan-PCR
1	Ad	delE746-A750	Wt	Wt	Wt
2	Ad	Wt	Wt	Wt	Wt
3	Ad	Wt	Wt	Wt	Wt
4	Ad	Wt	Wt	Wt	Wt
5	Ad	Wt	delL747-T751insP*	L858R	L858R
6	Ad	Wt	Wt	L858R	L858R
7	Ad	delE746-A750	delE746-A750	L858R	L858R
8	Ad	Wt	Wt	L858R	L858R
9	Ad	delE746-A750	delE746-A750	L858R	L858R
10	Ad	Wt	delL747-P753insS*	Wt	Wt
11	Ad	Wt	Wt	L858R	L858R
12	Ad	Wt	Wt	L858R	L858R
13	Ad	Wt	Wt	L858R	L858R
14	Ad	Wt	Wt	L858R	L858R
15	Ad	Wt	Wt	L858R	L858R
16	Ad	delE746-A750	delE746-A750	L858R	L858R
17	Ad	Wt	Wt	L858R	L858R
18	Ad	Wt	Wt	L858R	L858R
19	Ad	Wt	Wt	L858R	L858R
20	Ad	Wt	Wt	L858R	L858R
21	Ad	Wt	Wt	L858R	L858R
22	Ad	Wt	Wt	L858R	L858R
23	Ad	Wt	Wt	L858R	L858R
24	Ad	Wt	Wt	L858R	L858R
25	Ad	Wt	Wt	L858R	L858R
26	Ad	Wt	Wt	L858R	L858R
27	Ad	Wt	Wt	L858R	L858R
28	Sq	Wt	Wt	L858R	L858R
29	Sq	Wt	Wt	L858R	L858R
30	Sq	Wt	Wt	L858R	L858R
31	Sq	Wt	Wt	L858R	L858R
32	Sq	Wt	Wt	L858R	L858R
33	Sq	Wt	Wt	L858R	L858R
34	Sq	Wt	Wt	L858R	L858R
35	Sq	Wt	Wt	L858R	L858R
36	Sq	delE746-A750	delE746-A750	L858R	L858R
37	Sq	Wt	Wt	L858R	L858R
38	Sq	Wt	Wt	L858R	L858R
39	Sq	Wt	Wt	L858R	L858R
40	Sq	Wt	Wt	Wt	Wt

Ad, adenocarcinoma; Sq, Squamous Cell Carcinomai; Del, deletions; Wt, wild type; ^*^Rare exon 19 deletion mutations.

**Figure 3:**
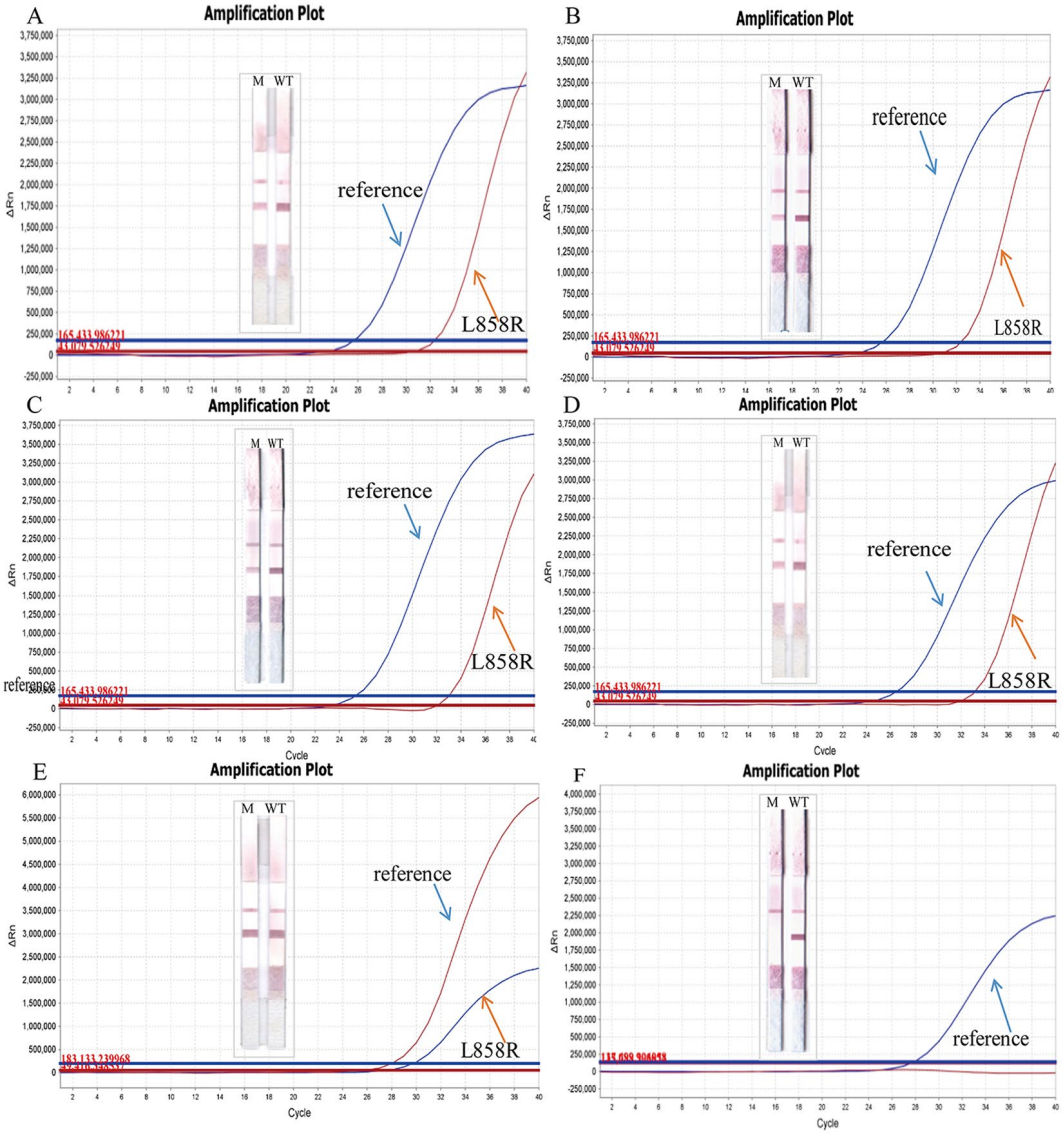
Results of L858R mutation detection by TaqMan-PCR method. Results were consistent with PCR-GoldMag LFA method. (**A**,**B**,**C**,**D**) representative samples were determined to be as L858R point mutation of *EGFR* by TaqMan-PCR. (**E**) Cell lines NCI-H-1975 were determined to be as L858R point mutation of *EGFR* by TaqMan-PCR. (**F**) The DNA control from whole blood collected from healthy individuals was determined to be wild-type *EGFR* by TaqMan-PCR.

**Figure 4:**
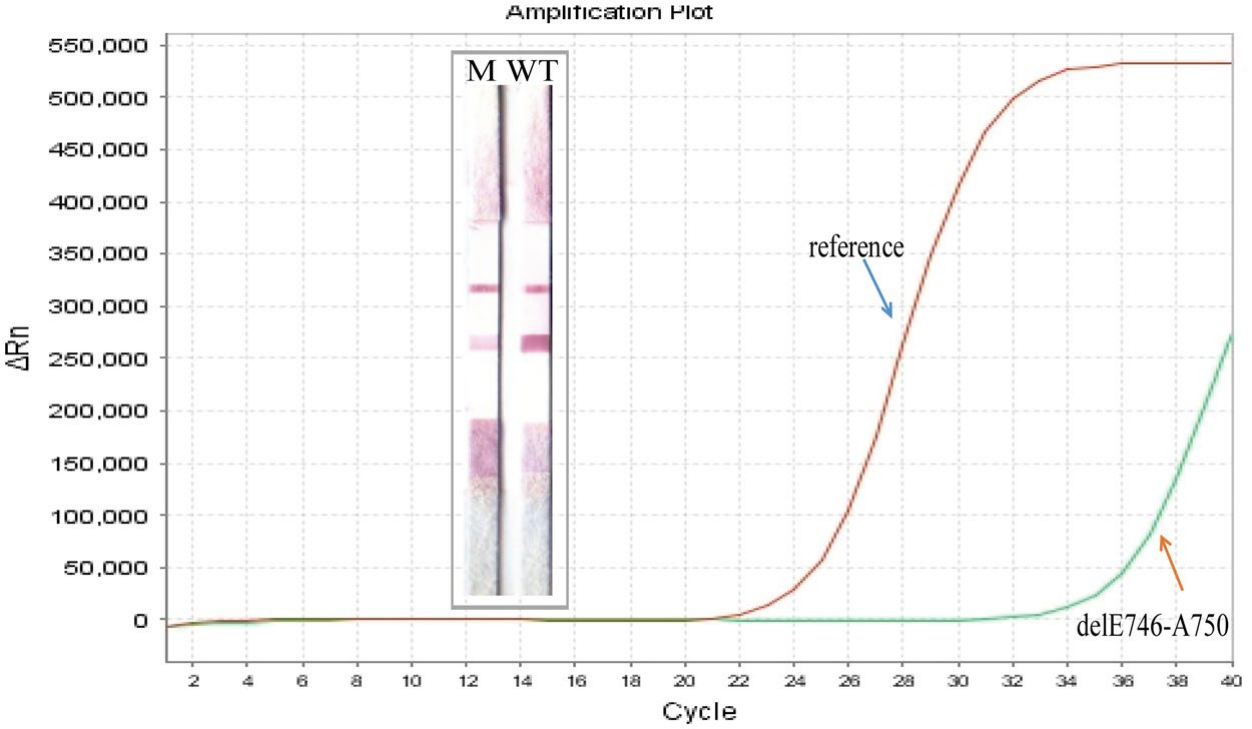
Validation the delE746-A750 result by qPCR method for No. 1 sample.

As shown in Table 2, direct sequence was compared with PCR-GoldMag LFA method, the statistical data showed that there was one discrepant sample with positive PCR-GoldMag LFA finding of delE746-A750 in exon 19, whereas DNA sequencing failed to detect the mutation. This might be because the sample contained low percentage of tumor cells. L858R mutation was validated via TaqMan-PCR method, which has been already reported to be a rapid and sensitive method for the detection of *EGFR* mutations^23^. Results of TaqMan-PCR assay agreed with our method, which showed 100% correspondence with the PCR-GoldMag LFA (show in Table 3, Fig. 3). The genotype of No.1 sample (discrepant with sequencing) was further confirmed by qPCR method, and the results showed that it harbors E746-A750 deletion mutation (Fig. 4), which was completely consistent with the PCR-GoldMag LFA method. Data indicated that PCR-GoldMag LFA was comparable to the TaqMan-PCR detection method, which was a novel approach for genotyping DNA mutations.

**Table 2:** Genotyping for E746-A750 by PCR-GoldMag LFA and DNA sequencing (n = 40).

PCR-GoldMag LFA	DNA sequencing			Total
	E746-A750	Other Deletions	Wt	
Positive	4	0	1	5
Negative	0	2	33	35
Total	4	2	34	40

**Table 3:** Genotyping for L858R by PCR-GoldMag LFA and TaqMan-PCR (n = 40).

PCR-GoldMag LFA	TaqMan-PCR		Total
	L858R	Wt	
Positive	34	0	34
Negative	0	6	6
Total	34	6	40

## Discussion

In this study, a new method for detecting the *EGFR* mutations including short deletion and SNP was established via PCR-GoldMag LFA using our patented gold magnetic nanoparticles (GoldMag) as a carrier. Our method showed excellent specificity and sensitivity results for mutant-specific detection versus direct sequencing. Moreover, it is comparable to other PCR-based methodologies, which are frequently used in the modern laboratory practice.

To confirm the results of PCR-GoldMag LFA, sequencing analysis was performed. Since direct sequencing is considered as the “gold standard” and many studies have reported it as a novel methodology^24–26^. However, our results showed that one sample was not checked by sequencing, which might be due to lack of sufficient number of mutant tumor cells for extraction of high-quality DNA of the sample. The choice of sequencing analysis method was subjected to the presence of more than 25% tumor cells of the samples^27, 28^. However, the sample could be detected by our method. These results confirm that our method was more sensitive than the direct PCR sequencing for clinical sample screening.

Current oncogene mutation detection technologies are expensive and time-consuming, and require complicated steps that were not routinely used in general hospitals or clinical laboratories. Therefore, a highly sensitive and fast assay is critical in this case. Compared to other proposed methods for the detection of *EGFR* mutant states in lung cancer, our PCR-GoldMag LFA detection method do not need special equipment other than thermocyclers, reaction steps are simple and allow troubleshooting, reagents are inexpensive and the methodology can be easily implemented at a clinical laboratory. After PCR, lateral flow device was used to detect the PCR products. It takes only 5 min to obtain the detection results without the need of expensive or high-end instruments. The whole process for detection of *EGFR* mutant states could be analyzed in less than two hours. We also demonstrated a high sensitivity of this method, with the use of different dilutions of DNA from tumor cell lines containing mutations in the *EGFR* gene. Results showed that we could detect activating mutations of the *EGFR* gene in specimens containing at least 0.78% of mutant DNA. Therefore, this method can be used in the laboratories of all levels of hospitals and medical institutions, especially in laboratories with limited resources.

The limitations of our study focused on using PCR-GoldMag LFA to detect *EGFR* mutations L858R and E746-A750 deletions in exon 19, but did not include other uncommon *EGFR*-TKI sensitive mutations, as this is a prospective study with primers that were specifically designed for these uncommon mutations are warranted in our laboratory. Secondly, our study included small sample size of NSCLC patient’s, and requires a larger sample size in the future studies.

One novelty of this method is that the PCR-GoldMag LFA using our patented GoldMag as a carrier for the first time detected small deletions, especially in tumor. Our method has been successfully applied to detect some single nucleotide polymorphisms, such as C677T on *MTHFR* and *ApoE* genotyping^22^. Herein, we also detected the small insertions/deletions in *EGFR* gene by our method. So, introduction of this method into clinical practice may open new opportunities for diagnosing as well as treatment of diseases that are caused by small insertion/deletion mutations.

## Conclusion

In summary, we have shown that the PCR-GoldMag LFA method can effectively detect the *EGFR* mutations, providing crucial information for diagnosis and therapeutic guidelines in NSCLC. Based on the results, our method can detect EGFR mutations with a limit of 0.78% in a heterogeneous DNA mixture. The simplicity of the present approach is that it can be easily implemented in a clinical laboratory, and offers a convenient and sensitive way over conventional methods.

## Materials and Methods

### Materials and reagents

GoldMag nanoparticles and lateral flow devices were provided from Xi’an GoldMag Nanobiotech Co., Ltd. (Xi’an, Shaanxi, China). 10 × HotMaster Taq Buffer and HotMaster Taq DNA Polymerase were purchased from TIANGEN (Beijing, China). dNTPs and uracil-DNA glycosylase (UDG) were obtained from Shinegene (Shanghai, China). Water (18.2 MΩ cm) used for all work in this report was purified by Barnstead Nanopure Water system. 1x TE (pH = 8) was purchased from Sangon Biotech. All primers were synthesized by Invitrogen (Shanghai, China). All tissue culture materials were obtained from Solarbio (Beijing, China).

### Positive and negative control samples

Whole blood was collected from healthy individuals and the genomic DNA was extracted as control for the development of PCR assays. Heterozygous control DNA was extracted from lung cancer cell lines, NCI-H-1650 and NCI-H-1975 (purchased from Procell, Wuhan, China), which contain the heterozygous delE746-A750 mutation in exon 19 and L858R mutation in exon 21, respectively. Wild-type DNA was extracted from the NCI-A549 cell line (purchased from Procell, Wuhan, China). The cell lines were cultured in RPMI 1640 medium supplemented with 10% fetal bovine serum (GE Healthcare Hyclone, United Kingdom). All cells were maintained in 5% CO_2_ at 37 °C (Thermo Forma; America).

Genomic DNA from whole blood or cell lines was extracted using whole blood genomic DNA isolation kit from Xi’an GoldMag Nanobiotech Co., (Xi’an, Shanxi, China) according to the manufacturer’s instructions.

### Tumor samples and DNA isolation

Tissue samples of this study were obtained from 40 patients diagnosed with lung cancer (NSCLC). The study protocol had been reviewed and approved by Ethical Review Committee of the First Affiliated Hospital of Xi’an Jiaotong University. All methods were performed in accordance with the approved guidelines. All the selected patients gave their consent for this study. Genomic DNA was isolated from 10 mm slices of formalin-fixed, paraffin-embedded (FFPE) section using FFPE DNA extraction kit (OMEGA, America) according to the manufacturer’s instructions. The concentration of DNA was analyzed by UV-Vis spectrometer (NanoDrop 2000, Thermo, America).

### The primers design for L858R and delE746-A750 in *EGFR* gene

According to the principle of allele-specific PCR^29^ for L858R genotyping, the reverse primers were designed as the common primers, and the forward primers are allele specific since the nucleotide at their 3′terminus corresponds to the SNP site. (Table 4). To ensure the specificity of the primers, an additional mismatch at the penultimate or antepenultimate nucleotide of the 3′terminus of allele specific forward primers was introduced^30^. For testing and proving the specificity of allele-specific PCR primers, plasmid was constructed containing L858R homozygous mutation and the sequences of plasmid was confirmed by sequencing.

**Table 4:** Primer sequences and PCR parameter.

	Primer	Primer sequences (5′-3′)	Optimal prime concentration (μmol/L)	Amplicon Size(bp)
Exon 21	F(M)	5′-digoxin-ATGTCAAGATCACAGATTTTGGGGT-3′	2.5	152
	F(WT)	5′-digoxin-ATGTCAAGATCACAGATTTTGGGGG-3′	2.5	
	R	5′-biotin-CAATACAGCTAGTGGGAAGGCAG-3′	2.5	
Exon 19	19P	5′-digoxin-GTAACATCCACCCAGATCACTG-3′	1.25	AQ: 339 PB: 155
	19Q	5′-biotin- CTCACTCATCATGCGTGTCAAG -3′	1.25	
	19A	5′-digoxin-CCCGTCGCTATCAAGGAATTCA-3′	2.5	
	19B	5′-biotin-CTTGTTGGCTTTCGGAGATGTTTTG-3′	1.25	

**Figure 5:**
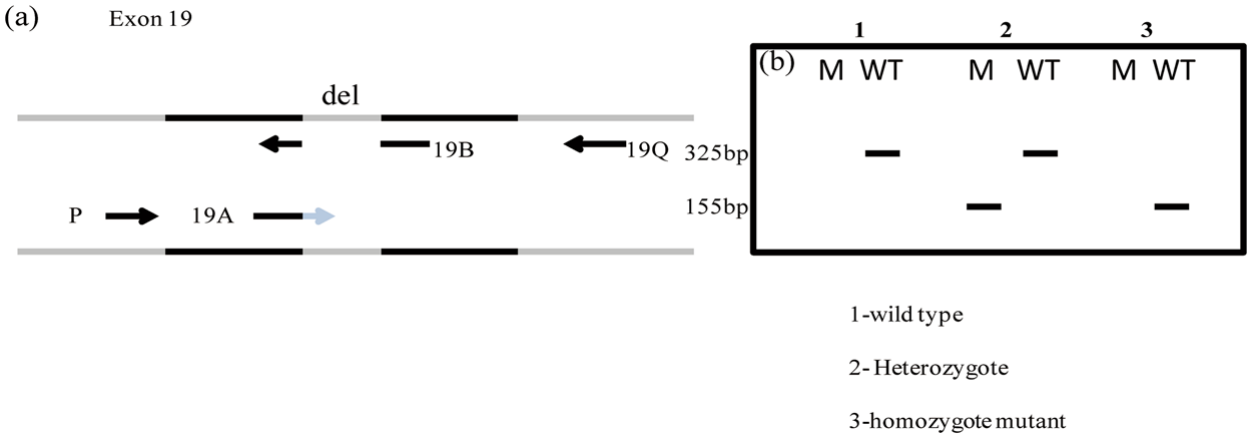
(**a**) Schematic illustration of designed Bi-PASA assay for the detection of exon 19 deletion. The deletion sequence variation is indicated by a light gray line. Two outer primers (P and Q) and two specific primers (A and B) are used to amplify specific products. Fragment sizes were: 155 bp for the deletion, 339 bp for the wildtype. (**b**). Predicted band pattern obtained with Bi-PASA genotyping. The AQ fragment can be amplified only with the wild-type sequence. PB can be amplified only in mutant DNA. A heterozygous genotype is characterized by both an AQ and PB amplification fragment. M = M tube. WT = WT tube.

For detecting the 15-bp deletion in exon19 of *EGFR* (codons 746–750), two tubes bi-directional PCR allele-specific amplification (Bi-PASA) assay was designed instead of the traditional one tube^31^, which can detect both possible nucleotide deletions (c.2235_2249del15 and c.2236_2250del15) that give rise to an E746-A750del protein change. The Bi-PASA reaction contains four primers: two specific primers: A and B, and two outer primers: P and Q (Table 4). The 3′end of inner primer A are complementary to a part of the deletion sequence and the deletion allele primer B are complementary to the mutant DNA sequence containing the deletion in opposite direction. The two outer primers (P and Q) were placed on the opposing strands at predefined bases. A two-tube (M tube and WT tube) Bi-PASA yields two overlapping fragments according to the genotype. AQ and PB are present in a heterozygote individual, whereas AQ is only produced in wild-type homozygote and PB only in homozygous mutant samples (Fig. 5). The two-tube PCR reactions allow distinction between different genotypes using the same DNA template. The M tube contains primer P and the deletion mutant primer B, intending to detect the deletion sequence PB. The WT tube contains primer Q and the inner primer A, aiming to detect the wild-type sequence AQ. Similarly, for testing and proving the specificity of the primers, plasmid was constructed containing delE746-A750 homozygous mutation and the sequence of plasmid was confirmed by sequencing.

### PCR amplification

The PCR reactions were performed at a final volume of 50 μL. Reactions consisted of genomic DNA with the concentrations of 50–100 ng/μL, 10 × PCR buffer (10 mM Tris HCl, 50 mM KCl), 200 μM of each dNTPs (dATP, dCTP, dGTP, dUTP), Hotmaster Taq DNA polymerase (0.5 U), UDG polymerase (0.5 U), and 1.25 μM-2.5 μM of primers. Cycling conditions were as follows: 50 °C for 2 min and 95 °C for 5 min, 32 cycles of 95 °C for 30 s, 60 °C for 30 s, 65 °C for 1 min, and a final extension at 65 °C lasts for 10 min.

### PCR-GoldMag based-LFA analysis

The 3 × 60 mm lateral flow strip is made up of five sections and includes a sample pad, a conjugate pad, a nitrocellulose (NC) membrane, an absorbent pad and a plastic backing. Some elements were treated before the device was constructed. Goat anti-mouse IgG and streptavidin were immobilized onto a porous nitrocellulose membrane (NC) respectively to form a control line (C-line) and a test line (T-line) by a BioJet (HM3010, BioDot Inc.). Next, the probe solution containing PAA (poly-acrylic acid) modified gold magnetic nanoparticles (PGMNs) conjugated with anti-digoxin antibody was dispensed onto the conjugate pad of PCR-GoldMag LFA strips. These strips were placed in a card box and stored in a sealed aluminum foil bag with desiccant silica gel at room temperature. The strips remain stable for 12 months. After PCR amplification, the whole PCR products in two tubes were added onto the sample pad, respectively and the results were read within 5 min visually. The reference DNA samples confirmed by direct sequencing were used to validate the method. The sensitivity was evaluated by serial dilution of the heterozygous mutant cell line with wild type cell line. The principle of PCR-GoldMag lateral flow device for the detection of delE746-A750 and L858R in NSCLC patients are similar as reported in our previous work^21^.
